# Genomic profiling of ovarian clear cell carcinoma in Chinese patients reveals potential prognostic biomarkers for survival

**DOI:** 10.1080/07853890.2023.2218104

**Published:** 2023-06-05

**Authors:** Shuang Ye, Shuling Zhou, Yutuan Wu, Xuan Pei, Wei Jiang, Wanling Shi, Wentao Yang, Xiaoyan Zhou, Boer Shan, Huijuan Yang

**Affiliations:** a Department of Gynecologic Oncology, Fudan University Shanghai Cancer Center, Shanghai, China; b Department of Oncology, Shanghai Medical College, Fudan University, Shanghai, China; c Department of Pathology, Fudan University Shanghai Cancer Center, Shanghai, China

**Keywords:** Ovarian clear cell carcinoma, next-generation sequencing, driver mutation, prognostic factor, prediction model

## Abstract

**Introduction:**

Ovarian clear cell carcinoma (OCCC) has distinct clinical and molecular features and heterogeneous prognosis. Insights into the somatic genomic abnormalities of OCCC provide the basis for deeper understanding and potential therapeutic avenues. Herein, we performed extensive genomic profiling in Chinese patients to illustrate the mutation landscape and genetic prognostic biomarkers of OCCC.

**Patients and methods:**

We used targeted DNA sequencing on 61 OCCC cases with a panel of 520 cancer-related genes. Correlations between clinicopathological features and survival were evaluated. Nomogram-based models were constructed to predict progress-free survival (PFS).

**Results:**

We detected 763 somatic mutations spanning 286 genes. The most frequent genetic alterations, ARID1A (49%) and PIK3CA (48%), were concurrently mutated. Comprehensive copy number alterations (CNAs) were identified in chromosomes 20q13.2 and 8q. Most (73.7%) patients harboured potentially targetable driver mutations. The mean and median tumour mutational burden were 7.0 and 3.0 mutations/Mb, respectively. Microsatellite instability (high) was identified in 8.2% of patients. Mutation of the base-excision repair pathway was significantly higher in patients of stage II/III/IV. ATM mutation was associated with platinum sensitivity (*p* < .05). Survival analysis identified chr8q CNAs in all patients, PIK3CA mutations in stage I patients and SWI/SNF complex (ARID1A and SMARCA4) mutations in stage II/III/IV patients as potential prognosticators (*p* < .05). Integration of genetic alterations (SWI/SNF complex mutations, ATM mutations and chr8q CNAs) improved the performance of a nomogram based on tumour stage and residual disease (concordance index 0.75 vs. 0.70, *p* < .05).

**Conclusions:**

We described somatic genomic alterations in Chinese OCCC patients and observed different genomic alterations between stage I and stage II/III/IV tumours. Genetic factors may supplement clinical factors in nomogram modelling for PFS prediction.

**Key Messages**
1. We performed extensive genomic profiling in a well-annotated cohort of 61 Chinese ovarian clear cell carcinoma (OCCC) patients.
2. PIK3CA mutations were associated with worse overall survival (OS) in stage I OCCC, and SWI/SNF gene mutations were associated with improved OS in stage II/III/IV disease.
3. We propose an easy-to-use nomogram using clinical factors (tumour stage and residual disease) and genetic alterations (SWI/SNF complex mutations, ATM mutations and chr8q CNAs) to predict the progress-free survival (PFS) of OCCC.

## Introduction

Ovarian cancer is a heterogeneous disease with different subtypes including high-grade serous carcinoma, low-grade serous carcinoma, mucinous carcinoma, endometrioid carcinoma and clear cell carcinoma [1]. Ovarian clear cell carcinoma (OCCC) has a wide geographical and racial distribution and is most prevalent among Asian women [2]. Compared with the other subtypes, OCCC has distinct clinical and molecular features [1,3]. We previously described the biological behaviour and clinicopathological features of Chinese OCCC patients [4–7]. OCCC patients are usually younger and diagnosed at earlier stages than those with the most common high-grade serous carcinoma [3]. Nonetheless, OCCC represents a great challenge and a largely unmet need given its disease aggressiveness and chemotherapy resistance [1,8].

The rapidly advancing clinical trials, targeted therapy, genetic testing and international collaborations have significantly changed the treatment landscape of ovarian cancer, mainly for patients with high-grade serous carcinoma [8]. There is currently no approved targeted agent for OCCC [8]. Several small proof-of-concept phase II clinical trials in OCCC have been reported with unsatisfactory results [9–11]. Deeper insights into the molecular background of OCCC undoubtedly facilitate the development of targeted therapeutics [8]. Endeavours have been undertaken to delineate the molecular features of OCCC in order to explore promising therapeutic targets from both genomic and transcriptomic levels [12–25]. The major findings of the mutational landscape of previous studies are summarized in Supplementary Tables S1 and S2. Of note, Friedlander et al. performed targeted sequencing with a 46-gene panel on tumour tissues from 105 Australian patients with pure OCCC histology. The authors identified PIK3CA (50%), TP53 (18.1%), KRAS (12.4%) among the frequently altered genes and moreover, 61% patients had at least one alteration in the PI3K/Akt/mTOR pathway components [12]. Aberrant ERBB2, PDGFRA and NRAS were each identified in one case. Itamochi et al. reported whole genome sequencing of tumour tissues from 55 Japanese OCCC patients [15]. Alterations to the SWI/SNF complex subunit, PI3K/Akt and receptor tyrosine kinase (RTK)/Ras pathways were found in 51%, 42% and 29% cases, respectively. Activated PI3K/Akt or RTK/Ras signalling pathways were suggested as independent prognostic factors. Elvin et al. [14] and Caumanns et al. [19] studied European and American cohorts using targeted sequencing and made similar observations. Altered in approximately 50% cases, PIK3CA and ARID1A were among the most frequently aberrant genes. Also, the majority of patients had an altered member on the PI3K pathway. In Chinese patients, Yang et al. reported altered ARID1A (64.3%), MUC4 (28.6%), PIK3CA (28.5%) and MAGEE1 (19%) identified by whole exome sequencing [25]. The aberrant genes were mostly involved in proliferation (PI3K/AKT, TP53 and ERBB2 pathways) and chromatin remodelling, with 83% and 71% patients harbouring at least one altered member, respectively. Given disease rarity, accumulating evidence and data on the aberrant genes and molecular pathways in OCCC might be the basis for a precise and personalized therapeutic approach.

In the current study, we performed extensive genomic profiling in a well-annotated cohort of 61 Chinese OCCC patients. The somatic genetic alterations and molecular pathways were identified and evaluated for association with clinicopathological features. Furthermore, we explored the potential biological prognostic biomarkers and tried to construct a prediction model for survival.

## Patients and methods

### Patients and samples

The study was approved by the Fudan University Shanghai Cancer Center Institutional Review Board (050432-4-1212B). It is a routine that patients scheduled for surgery are asked whether or not they are willing to donate their blood and/or tumour samples for research purposes in our institution. Blood samples are collected the day before surgery, while tumour samples are obtained during operation from those who provide informed consent.

We identified all the patients by searching the surgical pathology archives for ‘ovarian clear cell carcinoma’ from 2014 to 2018. The slides were reviewed by a gynaecology-dedicated pathologist (Dr. Shuling Zhou) to confirm the diagnosis and mixed histology was excluded. Then, the formalin-fixed paraffin-embedded (FFPE) tumour samples were collected. The matching peripheral blood or normal tissue was also obtained for each patient to exclude germline aberrations. After sample quality control, 61 samples were included for targeted sequencing, using a targeted panel of 520 cancer-related genes (OncoScreen Plus; Burning Rock Biotech, Guangzhou, China). The panel covers all exons of 312 genes and selected exons, introns or promoter regions of the remaining 208 genes, spanning 1.87 megabases of the human genome, including a total coding region size of 1.003 Mb. A detailed list of the 520 genes and the type of targeted sequencing can be found in Supplementary Table S3. The major exclusion criteria were: (1) mixed histology and (2) failure of quality control for genomic testing. Clinicopathological information and survival outcomes were retrospectively collected from medical records. Patients were considered as platinum-sensitive if the platinum-free interval (the time interval from completion of the last platinum-based chemotherapy to disease recurrence) was more than six months. Progression-free survival (PFS) and overall survival (OS) were defined as the time interval from the date of the primary surgery to the date of first recurrence and death or last contact, respectively. Written informed consent was obtained from each patient.

### DNA extraction and targeted sequencing

The QIAamp DNA FFPE Tissue Kit (Qiagen, Hilden, Germany) and QIAamp DNA blood Kit (Qiagen, Hilden, Germany) were used to extract DNA from tumour samples and non-tumour samples or white blood cells according to the manufacturer’s instructions. The Qubit 2.0 fluorometer and the Qubit dsDNA HS Assay Kit (Life Technologies, Carlsbad, CA) were used to measure DNA concentration.

The M220 Focused-ultrasonicator (Covaris, Woburn, MA) was used to shear DNA, followed by end repair, phosphorylation and adaptor ligation. The Agencourt AMPure XP beads (Beckman Coulter, Brea, CA) were used to select DNA fragments with the range of 200–400 bp. Then, hybridization with capture probe baits, hybrid selection with magnetic beads and PCR amplification were performed. Target capture was performed with commercially available panel of 520 genes (OncoScreen Plus, Burning Rock Biotech, Guangdong, China). DNA quality and fragment size were assessed by Bioanalyzer 2100 (Agilent, Santa Clara, CA). The indexed samples were sequenced on Illumina NextSeq 500 paired-end system (Illumina, Inc., Hayward, CA).

### Sequencing data analysis

Sequence data were analysed as we previously reported [26]. Copy number alteration (CNA) was called if the coverage data of the gene region was quantitatively and statistically significant from its reference control. The limit of detection for CNV is 1.5 for deletions and 2.64 for amplification. Tumour mutational burden (TMB) was calculated based on the ratio between the count of nonsynonymous mutations and the total size of coding region of the gene panel. Microsatellite instability (MSI) status was determined by the percentage of unstable loci through a read-count-distribution-based approach [27].

### Survival analysis

The Kaplan–Meier method was used to estimate survival rate and plot survival curves. To assess the association between genomic alterations and PFS or OS, we used the log-rank test for univariate analysis and the Cox proportional hazards regression model for multivariate analysis.

### Statistical analysis

The continuous variables were presented as mean or median. The categorical variables were presented as frequencies. Unpaired Wilcoxon’s signed-rank test was used to compare continuous variables, while two-sided Fisher’s exact tests were used to compare categorical variables, as appropriate. *p* < .05 was considered statistically significant. All bioinformatics analyses were performed with R (v.3.5.3, the R Foundation for Statistical Computing, Vienna, Austria). The prediction models were constructed using nomogram [28].

## Results

### Patients’ characteristics

Table 1 shows the clinicopathological features of the study cohort. The most majority (56/61) patients were primary cases and five were recurrent. The median age of the OCCC patients was 52 years (range, 26–79 years). Of all, 50.8% (31/61) were at stage I, 16.3% (10/61) were at stage II, 23.0% (14/61) were at stage III and 8.2% (5/61) were at stage IV and one patient with unknown stage (1.6%). A total of 49 patients (80.3%) had no gross residual disease after primary surgery. In terms of platinum response, platinum-resistant and platinum-sensitive recurrence account for 29.5% (18/61) and 68.9% (42/61), respectively. During the study period, recurrence and death were observed in 54.1% (33/61) and 36.1% (22/61) of the patients, respectively.

**Table 1.** Patients’ characteristics.

Median age (range) (years)	52 (26–79)
Tumour stage	
Stage I	31 (50.8%)
Stage II	10 (16.3%)
Stage III	14 (23.0%)
Stage IV	5 (8.2%)
Unknown	1 (1.6%)
Residual tumour	
No residual disease	49 (80.3%)
With residual disease	10 (16.4%)
Unknown	2 (3.3%)
Platinum response	
Platinum sensitive	42 (68.9%)
Platinum resistant	18 (29.5%)
Unknown	1 (1.6%)
Recurrence	
Yes	33 (54.1%)
No	26 (42.6%)
Unknown	2 (3.3%)
Death	
Yes	22 (36.1%)
No	38 (62.2%)
Unknown	1 (1.6%)

### Genomic landscape of OCCC

**Figure 1.**
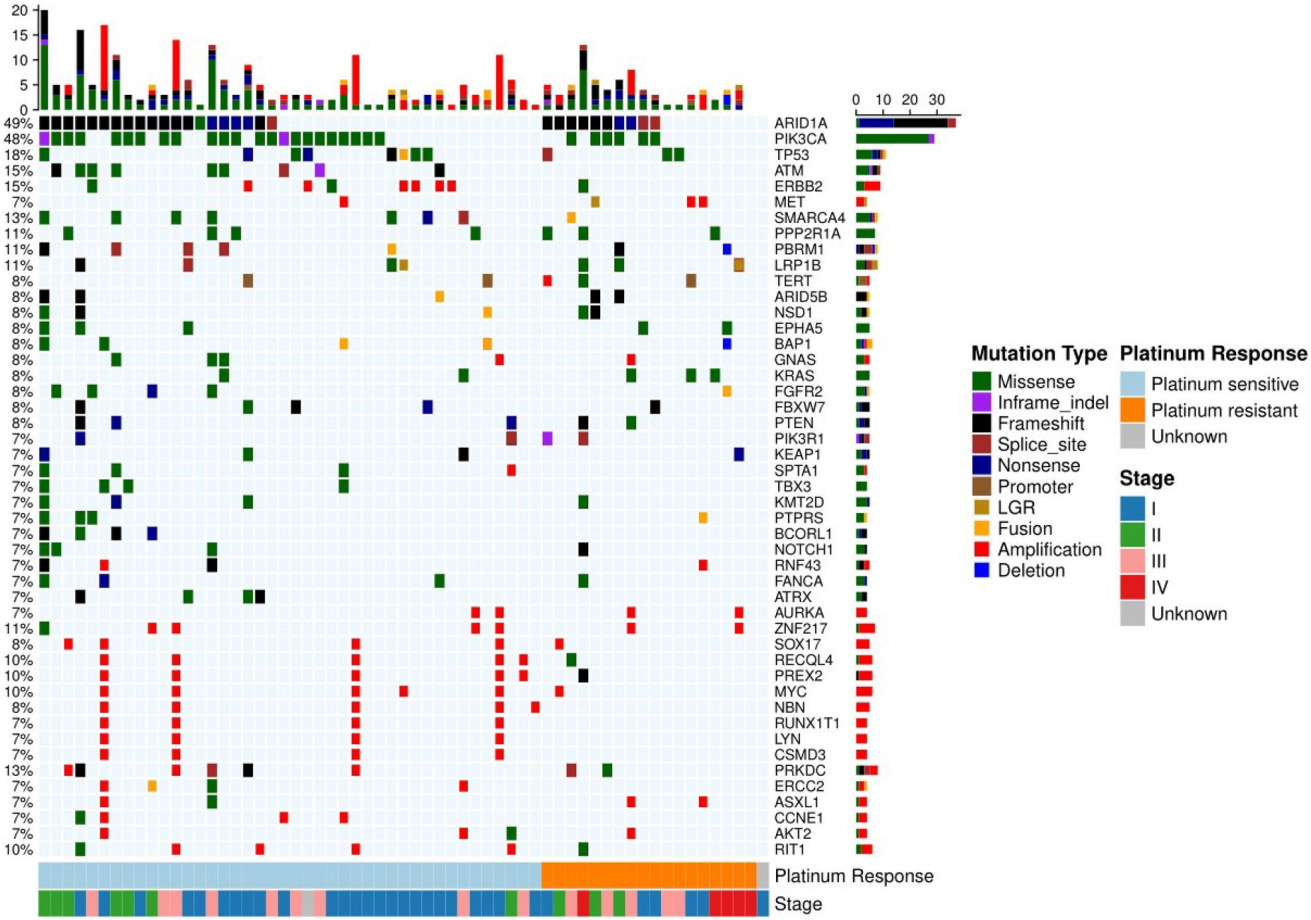
An oncoplot view showing the somatic genomic alterations in the most frequently altered genes. Each column represents a tumour sample and each row represents a gene analysed with targeted sequencing. The upper plot shows the frequency of mutation for each tumour sample. The central plot shows the types of mutations in each tumour sample (when the sample showed more than one mutation in the same gene, only the most deleterious type is shown). The lower part of the figure shows the response to platinum therapy, tumour stage and immune status of each sample.

A total of 763 somatic mutations were detected, spanning 286 genes in 61 patients. Distributions of somatic alterations are presented in Figure 1. More details of the alterations are provided in Table S4. The most common mutant genes were ARID1A (49%) and PIK3CA (48%). Mutations were also frequently observed in other genes, including TP53 (18%), ATM (15%), SMARCA4 (13%) and PRKDC (13%). Among the 30 patients harbouring ARID1A alterations, 20 patients had frameshift mutations, six patients with nonsense mutations, three patients with splice site mutations and one patient with a missense mutation. The majority (93.1%, 27/29) of the PIK3CA mutations were missense mutations. Interestingly, concurrent mutations of ARID1A with PIK3CA and PRKDC were observed, respectively. To be more specific, 20 (32.8%) patients had coexistent mutations of ARID1A and PIK3CA while seven (11.5%) patients had concurrent alterations of ARID1A and PRKDC. No alterations were identified in BRCA1/2. CNAs in 8q and 20q13.2 were frequently detected in our cohort. There were nine (14.8%) patients harbouring CNAs in chromosome 8q covering genes including PRKDC, RECQL4, PREX2, SOX17, MYC, NBN, RUNX1T1, LYN and CSMD3, and six (9.8%) patients harbouring CNAs in chromosome 20q13.2 covering AURKA and ZNF217.

**Figure 2.**
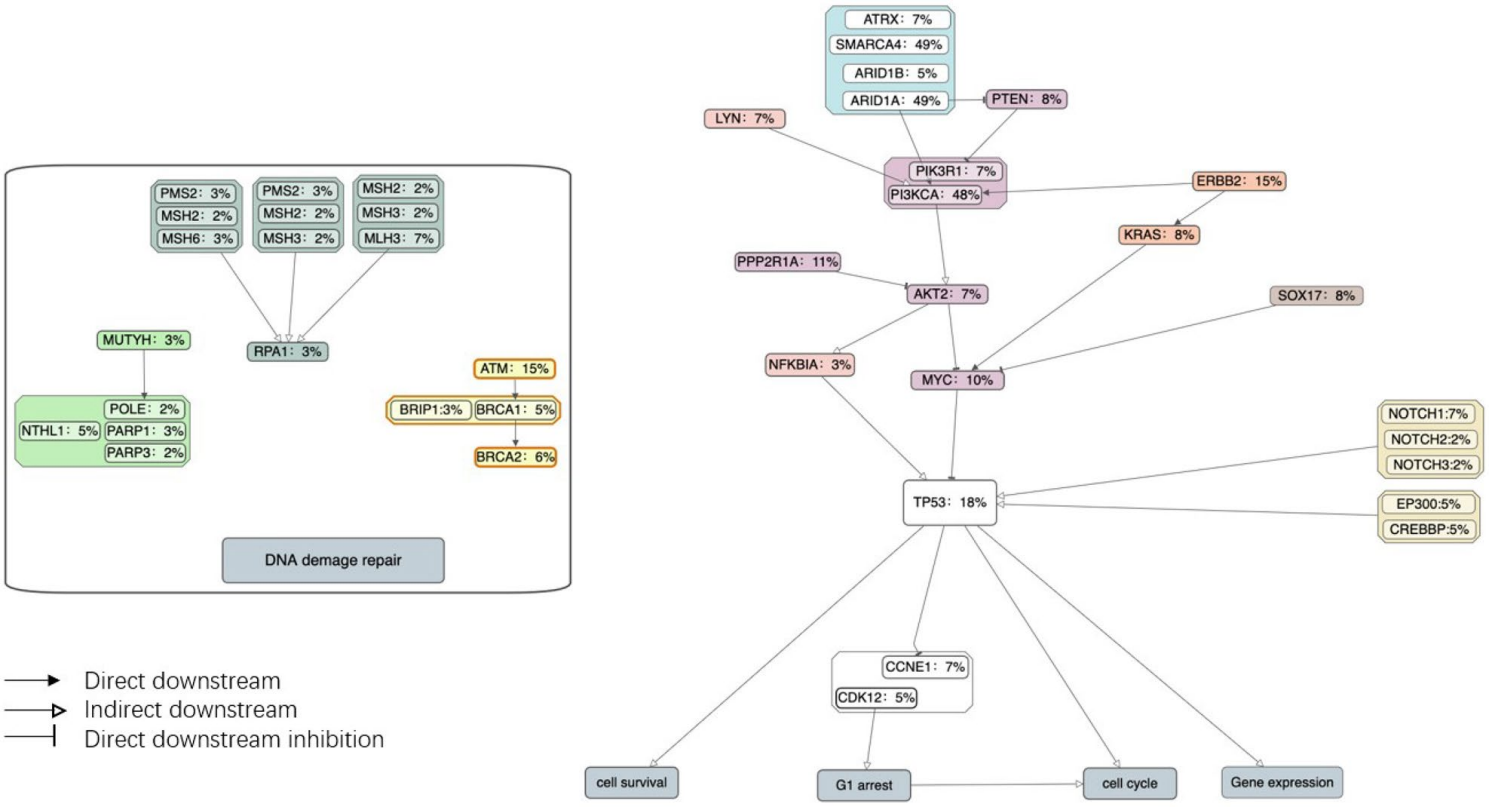
Pathways and known interactions between aberrant genes, clustered by pathway. Genes belonging to the same pathway were indicated with same colour. The percentages following the gene names indicate frequency of genomic alteration in our cohort. The arrows indicate the type of interaction (i.e. directly downstream, indirectly downstream and directly inhibitory) between different genes within a pathway.

Pathway analysis identified a large number of mutations across several major pathways: PI3K-Akt (60.7%), SWI/SNF complex (54.1%), HRR (29.5%), ErbB signalling (21.3%), MMR (18.0%), NF-kB signalling (16.4%), BER (13.1%), Wnt signalling (11.5%) and Notch signalling (11.5%). The mutation landscape of these major pathways with mutation rates of relevant genes are indicated in Figure 2.

We next evaluated potentially actionable driver mutations, which were identified in 45 patients (73.7%) (Supplementary Figure S1). The most common driver mutations were missense mutations in PIK3CA (64%). Other driver mutations, such as alterations in PTEN, ERBB2 and FGFR of the PI3K pathway, MET and EGFR of the RTK pathway, and KRAS of the ERK/MEK pathway, were also reported.

### Tumour mutational burden and microsatellite instability

The mean and median TMB of the study population were 7.0 and 3.0 mutations/Mb, respectively. MSI-high (MSI-H) was observed in five (8.2%) patients. TMB levels of each patient were presented in relation to MSI and mutation status of major pathways (Supplement Figure S2A). Among five patients with MSI-H, four had TMB ≥ 20 mutations/Mb. Another MSI-H patient and a patient with concurrent mutations in SWI/SNF complex/HRR/BER pathway also showed high TMB levels though less than 20 mutations/Mb (Supplementary Figure S2A). Further comparisons of TMB and MSI with different genes and pathways indicated that mutations of SWI/SNF complex, DNA damage response (DDR) pathway, and NOTCH1 were associated with higher TMB level and MSI proportion (Supplementary Figure S2B and S2C).

### Associations between somatic genomic alterations and clinicopathological features

**Figure 3.**
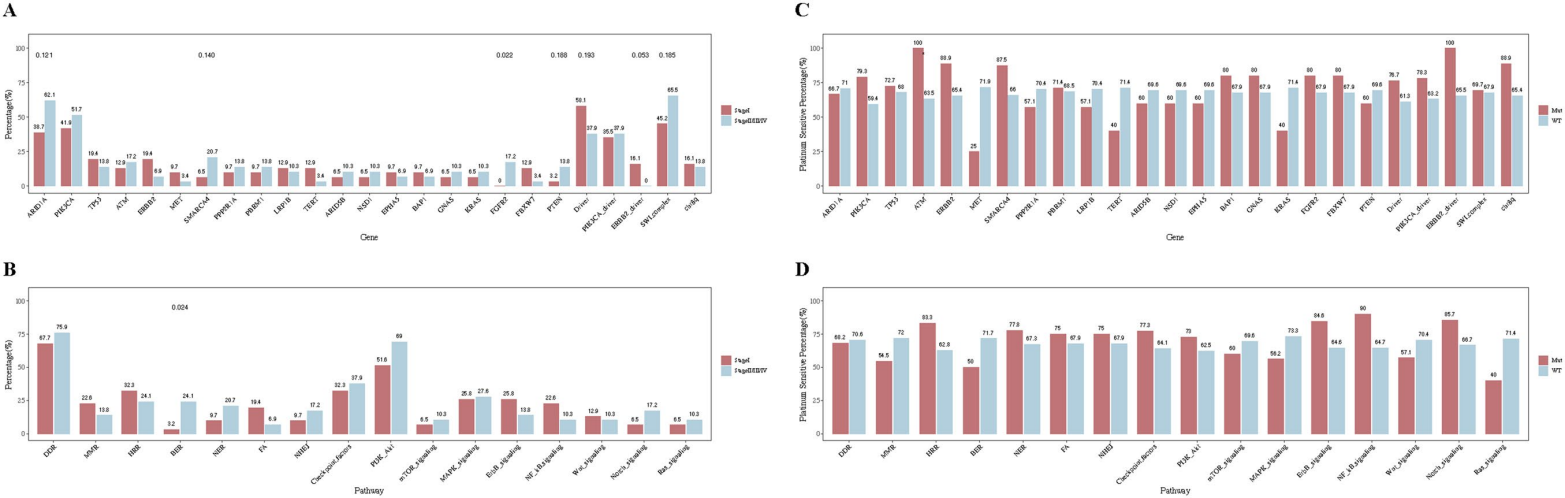
Distribution of somatic genetic alterations in patients with tumours of different stages or platinum sensitivity. (A) Frequencies of different alterations in patients of with stage I or any of stage II, III and IV disease. (B) Frequencies of pathways that carried ≥1 alteration in patients with stage I or stage II/III/IV disease. (C) Percentages of platinum-sensitive patients in all who harboured the indicated alteration. (D) Percentages of platinum-sensitive patients in all who harboured ≥1 aberrant gene participating in the indicated pathway.

Next, we evaluated the relationships between somatic genomic alterations and clinicopathological parameters including tumour stage and platinum response. In the first step, the distribution of mutations in genes and pathways was compared between patients with stage I disease and stage II/III/IV disease (Figure 3(A,B)). Interestingly, 17.2% of the patients with stage II/III/IV tumours had FGFR2 mutation while no FGFR2 mutation was found in the stage I group (*p* < .05). Mutations in ARID1A and PIK3CA occurred more frequently in stage II/III/IV than in stage I (ARID1A: 62.1% vs. 38.7%, PIK3CA: 51.7% vs. 41.9%), though statistical significance was not achieved. On the contrary, patients with stage I disease tended to have a higher mutation rate of ERBB2/HER2 (19.4% vs. 6.9%) and MET (9.7% vs. 3.4%), albeit with no statistical significance. When it comes to pathway, mutations in the BER pathway, which belongs to DDR, were more frequently presented in stage II/III/IV than stage I (24.1% vs. 3.2%, *p* < .05). Consistent with gene mutations, the DDR and PI3K pathway alterations were more commonly observed in patients with stage II/III/IV than stage I counterparts though significance was not achieved.

In the second step, we assessed the potential role of genomic alterations in platinum response (Figure 3(C,D)). Patients with ATM mutations had higher percentage of platinum-sensitive recurrence (100% vs. 63.5%, *p* < .05). No other significant correlations were found between gene and/or pathway alterations and platinum response.

### Prognostic implications of genomic alterations

We further investigate the role of genetic alterations as potential prognostic biomarkers by evaluating the association between individual alterations and OS or PFS with the log-rank test. Alterations significantly (*p* < .05) associated with survival outcomes were considered as potential prognosticators. For OS, 81.6% (25/31) and 48.3% (14/29) were censored among stage I and stage II/III/IV patients, respectively. For PFS, 61.3% (19/31) and 31.0% (9/29) patients were censored among stage I and stage II/III/IV patients, respectively (Table S5). No significant difference in survival outcomes was observed between patients carrying mutated PIK3CA, ERBB2/HER2 or MET and their wild-type counterparts. Similarly, mutations of the SWI/SNF subunit genes did not correlate with survival outcomes. It is worth mentioning that patients harbouring CNAs in chr8q had significantly improved OS (*p* < .05).

**Figure 4.**
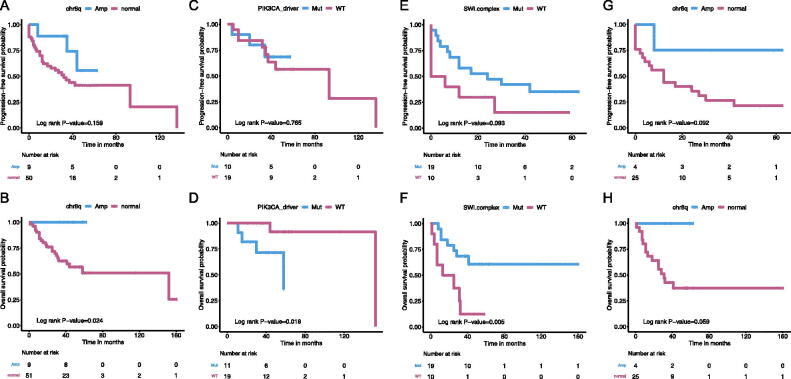
Kaplan–Meier’s survival curves of patients stratified by different genomic alteration and log-rank test evaluating the significance of indicated alteration with survival outcomes. The top panel showed the progression-free survival and the bottom panel the overall survival. (A, B) Survival curves of patients with or without chromosome 8q amplification. (C, D) Survival curves of stage I patients with or without driver mutations in *PIK3CA*. (E, F) Survival curves of patients harbouring ≥1 aberrant member of the SWI/SNF complex or their wild-type counterparts. (G, H) Survival curves of stage II/III/IV patients with or without chromosome 8q amplification.

Considering the heterogeneous prognosis of OCCC, further analyses were performed in patients with stage I and stage II/III/IV diseases, separately. Interestingly, PIK3CA driver mutations were significantly associated with worse OS (*p* < .05) and a trend of worse PFS in patients with stage I disease. In stage II/III/IV OCCC, log-rank analysis indicated that patients harbouring mutations of the SWI/SNF subunit genes showed significantly improved OS (*p* < .01) and the trend of better PFS. ARID1A and SMARCA4 are key members of the SWI/SNF complex. In patients with ARID1A mutations, significantly improved OS (*p* < .05) and the trend of better PFS were observed. The presence of SMARCA4 mutations was associated with significantly improved PFS (*p* < .01) and the trend of better OS. Patients with PIK3CA driver mutations showed comparable survival outcomes with wild types. Patients with chr8q CNAs tended to have better OS and PFS albeit with no significance (Figure 4). Then, multivariate analyses involving residual disease, stages and candidate genetic alterations were performed. Mutations in the SWI/SNF complex were shown to be an independent prognostic factor for both PFS (HR = 0.37, *p* < .05) and OS (HR = 0.22, *p* < .01) in patients with stage II/III/IV OCCC.

### Construction of nomogram for survival prediction

**Figure 5.**
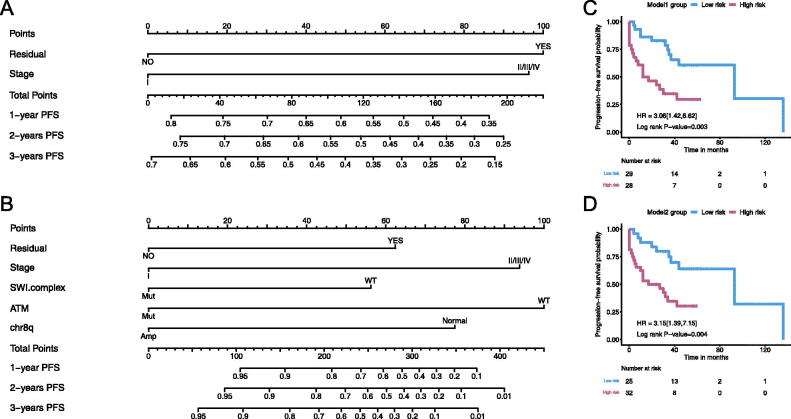
Nomogram models for predicting progression-free survival (PFS) of ovarian clear cell carcinoma based on (A) clinical and (B) clinical and molecular features. In both (C) model 1 and (D) model 2, patients in the high-risk group had poor progression-free survival than the low-risk group.

To reveal the significance of clinical factors and genetic factors in prognosis prediction, we sought to build prediction models for PFS in OCCC patients by nomogram based on our dataset (Figure 5). Only patients with known residual disease status (*n* = 59) were included in nomogram construction. Model 1 was built on clinical factors only. Residual disease (HR = 2.40) and groups of disease stage (stage II/III/IV vs. stage I, HR = 2.32) were used to predict 12-, 18- and 24-month PFS based on the total points of each factor. The concordance index (c-index) of model 1 was 0.70. To evaluate if genetic factors could further improve the predictive performance, we build model 2 using clinical factors combined with genetic alterations. In model 2, clinical factors included residual disease (HR = 2.42) and groups of disease stage (HR = 3.77), while genetic alterations included SWI/SNF complex mutations (HR = 0.45), chr8q CNAs (HR = 0.33) and ATM mutations (HR = 0.24). The c-index of model 2 was 0.75. Both model 1 and model 2 indicated that patients in the high-risk group had poor PFS than the low-risk group (model 1: HR = 3.06, *p* < .05, model 2: HR = 3.15, *p* < .05, Figure 5). Comparison of these two models indicated that the use of clinical factors with genetic alterations (model 2) showed better performance than clinical factors alone (model 1) in predicting PFS of OCCC (*p* < .05).

## Discussion

In this study, extensive genomic profiling was performed on a well-annotated cohort of 61 Chinese OCCC patients. We found PIK3CA mutations associated with worse prognosis in stage I patients and SWI/SNF gene mutations with improved prognosis in stage II, III or IV patients. Furthermore, we built an easy-to-use nomogram using both clinical (tumour stage and residual disease) and genomic factors (SWI/SNF complex mutations, ATM mutations and chr8q CNAs) to predict the PFS of OCCC.

Recently, the genomic alterations and molecular pathways of OCCC have been explored in quite a few studies (Supplementary Tables S1 and S2) [12–22,24,25,29,30]. Eight out of the 15 studies came from Japan [15–18,21,22,24,29]. Two publications focused on Chinese patients, comprising a total of 89 patients [25,30]. Here, we performed genomic profiling in a well-annotated group of 61 Chinese OCCC patients to investigate the mutation landscape and underlying clinicopathological implications. The most frequently altered genes were ARID1A (49%) and PIK3CA (48%), which were consistent with a study in Chinese patients using the same targeted panel, in which ARID1A and PIK3CA were mutated in 50% and 52% cases, respectively [26]. In another study of 74 Chinese patients, Yang et al. reported that ARID1A was altered in 54.0% and PIK3CA in 41.3% patients [25]. The lower rate for PIK3CA may have arisen from cohort characteristics. Yang’s cohort included a higher percentage of stage I patients (65.2%) than ours (50.8%). We also noted in our cohort that ARID1A and PIK3CA were less frequently mutated in stage I than in the remaining patients, thereby leading to less frequently aberrant PIK3CA. ARID1A, on the other hand, was altered at similar rates despite different percentages of stage I patients, suggesting other clinical factors associated with ARID1A mutation frequency. We also assess the MSI status and TMB levels in our patients. MSI-H was identified 8.2% of patients, similar to a previous study by Elvin et al. who reported 4% patients of MSI-H status in 125 advanced/recurrent OCCC cases [14]. TMB level was low, with mean and median levels of 7.0 and 3.0 mutations/Mb, respectively, which was consistent with a previous study of ours (mean 4.4) [26]. Using a 160-gene panel, Saotome et al. found TMB ≥10 mutations/Mb in 10% (3/30) OCCC cases [24]. Similarly, we found 9.8% (6/61) patients with TMB ≥10 (Figure S2A) despite a different targeted panel. This consistency supported the validity of the TMB estimation. Interestingly, five of the six patients had MSI-H and the remaining had aberrant member of the SWI/SNF chromating remodelling and DDR pathways, suggesting DNA regulation as potential therapeutic avenues for patient with high TMB. In terms of gene amplifications and losses, Kim et al. reported copy number gains or losses in individual genes such as NTRK1 amplification or TSC1 loss, but did not identify chromosome-level variations [20]. In comparison, our study led to the novel findings of frequent CNAs in 20q13.2 and 8q.

Different from ovarian high-grade serous carcinoma, more than 50% of OCCC patients were diagnosed with stage I tumours [3]. In our previous study, we found that early-stage OCCC patients had favourable PFS compared with serous counterparts [5]. However, OCCC patients with advanced tumours had significantly worse survival including both PFS and OS [5]. Therefore, we tried to investigate the genetic alterations and pathways based on stage stratification. BER signalling, one of the DDR pathways, showed a significantly higher mutation rate in stage II/III/IV OCCC patients than in stage I. Genes of the SWI/SNF complex (ARID1A and SMARCA4) and the PI3K pathway (PIK3CA and PTEN) showed higher mutation rates in stage II/III/IV patients than stage I, although the statistically significant threshold was not achieved, which might be due to relatively small sample sizes. Further investigations on the mutational landscapes of different disease stages could contribute to the understanding of OCCC at molecular level.

It is still not entirely elucidated how genomic alterations affect the clinical behaviour in OCCC [15] and few studies have evaluated the possible prognostic implications of gene and pathway alterations. Itamochi et al. performed whole-genome sequencing in 55 Japanese patients and suggested altered PI3K/Akt and RTK/Ras pathways as prognostic biomarkers for OCCC patients [15]. In our study, we noted that CNAs in chr8q was associated with improved OS in all patients. PIK3CA mutations were significantly associated with worse OS in stage I patients. In patients with stage II/III/IV diseases, mutations in the SWI/SNF complex (ARID1A and SMARCA4) were associated with better OS. Multivariate analysis with other clinical factors and genetic alterations revealed that stage II/III/IV OCCC patients with mutations in the SWI/SNF complex showed significantly improved PFS and OS. In addition to conventional factors such as tumour stage and residual disease, genetic alterations such as SWI/SNF complex mutations emerged as a novel class of prognostic marker for OCCC. The nomogram-based models also indicated that the combination of clinical and genetic prognostic markers was better than clinical factors alone in predicting PFS of 61 patients with OCCC. Given the limited sample size in our study and the lack of external cohorts with enough clinical and genetic information, validation of these models could not be performed. Future prospective studies are required to validate and optimize the model performance.

Therapeutic options for late-stage OCCC patients are still limited to conventional chemotherapies. The development of novel targeted therapies for OCCC remains an unmet clinical need. In the last decade, there have been great advances in understanding the molecular profile of OCCC [12–25,29,30]. Although numerous genetic alterations have been identified in OCCC, none of the targeted regimens have been approved yet [8]. Considering the high frequency of mutation, SWI/SNF complex (ARID1A and SMARCA4) and PIK3CA should be considered as the major therapeutic targets of OCCC. A recent review on our understanding of the pathogenesis of OCCC summarized the direct and synthetic-lethal therapeutic strategies, particularly on those targeting ARID1A mutation [8]. Sunitinib and cabozantinib, which are multi-kinase inhibitors targeting vascular endothelial growth factor receptors, showed minimal activity in the second- and third-line treatment in OCCC as a single agent [9,11]. Similarly, in another phase II trial, single-agent ENMD-2076 did not meet the preset bar for efficacy [10]. However, loss of ARID1A was associated with better PFS on ENMD-2076 [10]. To target the vulnerabilities exposed by ARID1A deficiency, synthetic lethal therapy had been considered as one of the major therapeutic strategies for OCCC. Inhibitors of different targets had been confirmed to have synthetic lethal relationship with ARID1A, such as PARP inhibitors, BET inhibitors, HDAC inhibitors and EZH2 inhibitors [31]. In our study, 45 of 61 (73.7%) patients harboured potentially actionable driver mutations in the PI3K pathway, ERK/MEK pathway and RTK pathway. In addition, mutations of the SWI/SNF complex, DDR pathway and NOTCH1 were associated with higher TMB levels and MSI, which have been recognized as biomarkers for immunotherapy. In a phase II trial of pembrolizumab for recurrent ovarian cancer, OCCC showed a trend of higher response rate than other subtypes [32]. In another phase II trial of nivolumab in 20 patients with platinum-resistant ovarian cancers, only two patients achieved durable complete response and one of them had OCCC [33]. Further clinical studies are warranted to evaluate the use of targeted therapies and immunotherapies for OCCC.

The study has several limitations. First, we only included 61 cases given the disease rarity. Second, some clinical information was incomplete due to the retrospective design. Third, the prognostic implication and nomogram lack external validation. Thus, more sequencing data will be necessary to help us further understand the molecular profile and biological behaviour of OCCC. Furthermore, a multi-omic study that integrates genomic and transcriptomic data would render a more comprehensive molecular depiction of OCCC [26,34]. Lastly, we have not verified the function of the mutated genes in OCCC or studied the mechanisms by which they promote tumour by biological experiment. Further research should be encouraged to gain mechanistic insights on the functions of the altered genes and pathways identified in this study.

## Conclusions

We performed target sequencing with a 520-gene panel in 61 Chinese OCCC patients. Patients with stage I tumour had different genomic alterations compared with those with stage II/III/IV tumour. PIK3CA mutations were associated with worse OS in patients with stage I disease, while SWI/SNF gene mutations were related to improved OS in patients with stage II/III/IV disease. We built a nomogram for PFS that merits further exploration.

## Supplementary Material

Supplemental Material

Supplemental Material

Supplemental Material

Supplemental Material

Supplemental Material

Supplemental Material

## Data Availability

The data that support the findings of this study are available from the corresponding author upon reasonable request.
