# Supplementary material for: Genomic profiling of ovarian clear cell carcinoma in Chinese patients reveals potential prognostic biomarkers for survival
Source: Ann Med. 2023 Jun 5;55(1):2218104. doi: 10.1080/07853890.2023.2218104 (PMC10243386; doi:10.1080/07853890.2023.2218104)
Supplement: Supplemental Material [file IANN_A_2218104_SM2780.docx]

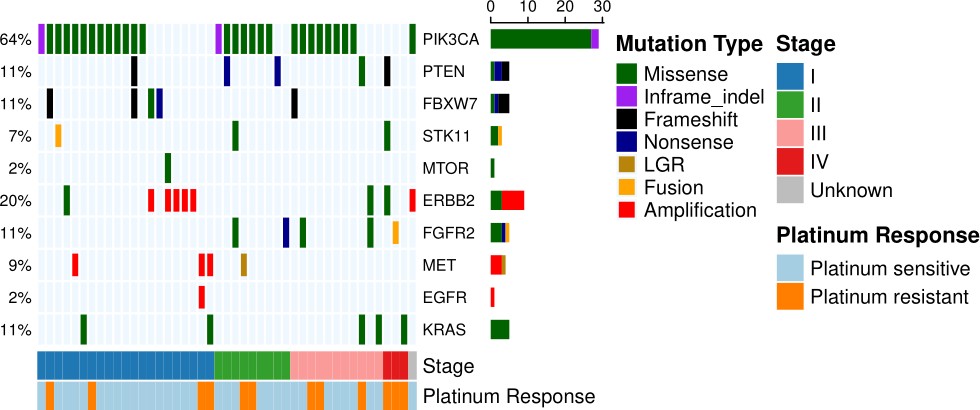


Supplementary Figure S1. Presence of potentially actionable driver mutations in 45 of 61 patients.


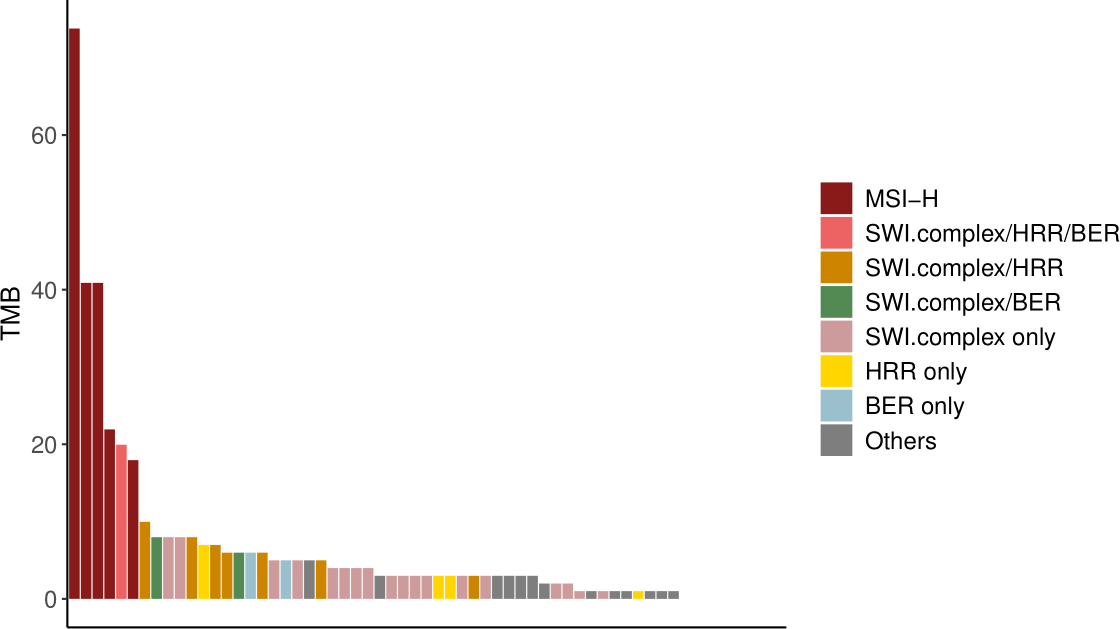
**S2A**

**S2B**


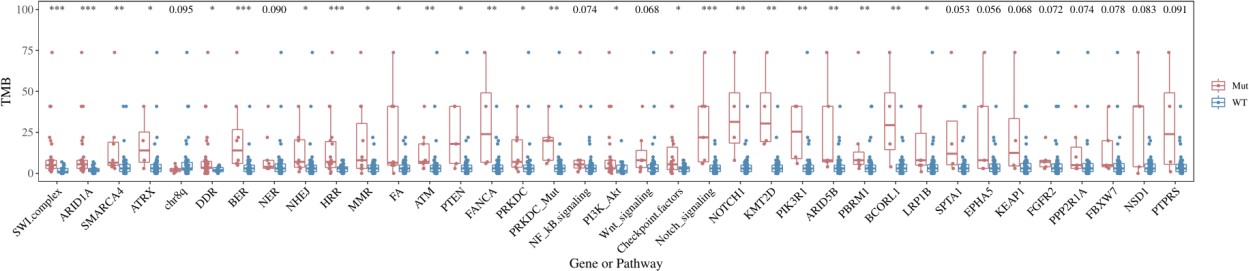


**S2C**


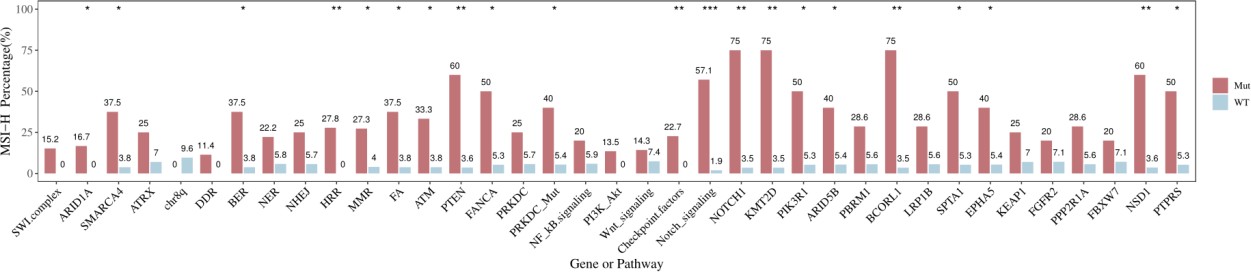


Supplementary figure S2. Associations of genetic alterations with tumor mutation burden (TMB) and microsatellite instability (MSI) in all stage patients. The distribution of TMB in patients with different MSI status and mutation status of major pathways (S2A). Comparison of TMB and MSI with different mutation status of genes and pathways.
