# Supplementary material for: Genomic profiling of ovarian clear cell carcinoma in Chinese patients reveals potential prognostic biomarkers for survival
Source: Ann Med. 2023 Jun 5;55(1):2218104. doi: 10.1080/07853890.2023.2218104 (PMC10243386; doi:10.1080/07853890.2023.2218104)
Supplement: Supplemental Material [file IANN_A_2218104_SM2779.docx]

Table S5. Number of patients with or without known recurrence or death status per stage.

|  | All (n=61) | Stage I (n=31) | Stage II/III/IV (n=29) |
| --- | --- | --- | --- |
| Recurrence |  |  |  |
| Yes | 33 (54.1%) | 12 (38.7%) | 20 (69.0%) |
| No | 26 (42.6%) | 17 (54.8%) | 9 (31.0%) |
| Unknown | 2 (3.3%) | 2 (6.5%) | 0 (0.0%) |
| Death |  |  |  |
| Yes | 22 (36.1%) | 6 (19.4%) | 15 (51.7%) |
| No | 38 (62.2%) | 24 (77.4%) | 14 (48.3%) |
| Unknown | 1 (1.6%) | 1 (3.2%) | 0 (0.0%) |
