# Supplementary material for: Genomic profiling of ovarian clear cell carcinoma in Chinese patients reveals potential prognostic biomarkers for survival
Source: Ann Med. 2023 Jun 5;55(1):2218104. doi: 10.1080/07853890.2023.2218104 (PMC10243386; doi:10.1080/07853890.2023.2218104)
Supplement: Supplemental Material [file IANN_A_2218104_SM2769.doc]

Supplementary Table S3. A list of the 520 cancer-related genes targeted by the OncoScreen Plus panel.

| ***ABL1*** | ***BCL2L1*** | ***CDKN2B*** | *EGFL7* | *FAS* | ***HIST1H3C*** | ***JAK1*** | ***MEF2B*** | ***NRG1*** | ***PMS1*** | ***RBM10*** | ***SOX2*** | *TRAF2* |
| --- | --- | --- | --- | --- | --- | --- | --- | --- | --- | --- | --- | --- |
| *ABL2* | *BCL2L11* | ***CDKN2C*** | ***EGFR*** | ***FAT1*** | ***HIST1H3D*** | ***JAK2*** | ***MEN1*** | ***NSD1*** | ***PMS2*** | *RECQL4* | ***SOX9*** | *TRAF7* |
| *ACVR1* | *BCL2L2* | ***CEBPA*** | *EIF1AX* | ***FAT3*** | ***HIST1H3E*** | ***JAK3*** | ***MET*** | ***NTHL1*** | *PNRC1* | *REL* | *SOX10* | *TRRAP* |
| *ACVR1B* | ***BCL6*** | *CENPA* | *EIF4A2* | ***FBXW7*** | *HIST1H3F* | ***JUN*** | *MGA* | ***NTRK1*** | ***POLD1*** | ***RET*** | *SOX17* | ***TSC1*** |
| *ADGRA2* | ***BCOR*** | ***CHD1*** | *EIF4E* | *FCGR2B* | ***HIST1H3G*** | *KAT5A* | ***MITF*** | ***NTRK2*** | ***POLE*** | *RFWD2* | ***SPEN*** | ***TSC2*** |
| ***AKT1*** | *BCORL1* | *CHD2* | *ELOC* | *FGF10* | ***HIST1H3H*** | ***KDM5A*** | ***MLH1*** | ***NTRK3*** | ***POM121L12*** | *RHEB* | ***SPOP*** | ***TSHR*** |
| ***AKT2*** | *BCR* | *CHD4* | ***EMSY*** | *FGF12* | ***HIST1H3I*** | ***KDM5C*** | ***MLH3*** | ***NUP93*** | *PPM1D* | *RHOA* | ***SPTA1*** | ***U2AF1*** |
| ***AKT3*** | ***BLM*** | ***CHEK1*** | ***EP300*** | *FGF23* | ***HIST1H3J*** | ***KDM6A*** | ***MPL*** | *PAK1* | ***PPP2R1A*** | ***RICTOR*** | ***SRC*** | ***VEGFA*** |
| ***ALK*** | *BMPR1A* | ***CHEK2*** | *EPCAM* | *FGF6* | *HIST2H3C* | ***KDR*** | ***MRE11A*** | *PAK3* | ***PPP2R2A*** | *RIT1* | ***SRSF2*** | *VEGFB* |
| *ALOX12B* | ***BRAF*** | *CHUK* | *EPHA2* | *FGF7* | ***HIST2H3D*** | ***KEAP1*** | ***MSH2*** | *PAK7* | *PPP6C* | ***RNF43*** | ***STAG2*** | *VEGFC* |
| ***AMER1*** | ***BRCA1*** | ***CIC*** | *EPHA3* | *FGFR14* | ***HIST3H3*** | ***KEL*** | ***MSH3*** | ***PALB2*** | ***PRDM1*** | ***ROS1*** | ***STAT3*** | ***VHL*** |
| *ANKRD11* | ***BRCA2*** | *CRBN* | *EPHA5* | *FGFR1* | ***HLA-A*** | ***KIT*** | ***MSH6*** | ***PARK2*** | *PREX2* | *RPA1* | *STAT4* | *VTCN1* |
| ***APC*** | ***BRD4*** | ***CREBBP*** | ***EPHA7*** | *FGFR2* | ***HNF1A*** | *KLF4* | *MST1* | *PARP1* | ***PRKAR1A*** | *RPS6KA4* | *STAT5A* | *WISP3* |
| *APCDD1* | ***BRIP1*** | ***CRKL*** | ***EPHB1*** | *FGFR3* | ***HNF1B*** | *KLHL6* | *MST1R* | *PARP2* | *PRKC1* | *RPS6KB2* | ***STAT5B*** | ***WRN*** |
| ***AR*** | *BTG1* | ***CRLF2*** | ***ERBB2*** | *FOXA1* | *HOXB13* | ***KMT2A*** | ***MTOR*** | *PARP3* | ***PRKDC*** | ***RPTOR*** | ***STK11*** | ***WT1*** |
| ***ARAF*** | ***BTK*** | ***CSF1R*** | ***ERBB3*** | ***FOXL2*** | ***HRAS*** | ***KMT2C*** | ***MUTYH*** | *PARP4* | *PRSS8* | ***RUNX1*** | *STK40* | *XIAP* |
| *ARFRP1* | *CALR* | *CSF3R* | *ERBB4* | *FRS2* | *HSD3B1* | ***KMT2D*** | ***MYC*** | ***PAX5*** | ***PTCH1*** | *RUNX1T1* | ***SUFU*** | ***XPO1*** |
| ***ARID1A*** | ***CARD11*** | ***CTCF*** | ***ERBB5*** | *FYN* | ***HSP90AA1*** | ***KRAS*** | ***MYCL*** | ***PBRM1*** | ***PTEN*** | *RYBP* | *SUZ12* | ***XRCC2*** |
| ***ARID1B*** | *CASP8* | *CTLA4* | ***ERCC1*** | *GABRA6* | *ICOSLG* | ***LATS1*** | ***MYCN*** | *PDCD1* | *PTK2* | ***SDHA*** | ***SYK*** | *XRCC3* |
| ***ARID2*** | ***CBFB*** | *CTNNA1* | *ERCC2* | *GATA4* | *ID3* | *LATS2* | ***MYD88*** | *PDCD1LG2* | ***PTPN11*** | *SDHAF2* | *TACC3* | *YAP1* |
| *ARID5B* | ***CBL*** | ***CTNNB1*** | *ERCC3* | *GATA6* | ***IDH1*** | ***LMO1*** | *MYOD1* | ***PDFRA*** | ***PTPRD*** | ***SDHB*** | *TAF1* | *YES1* |
| ***ASXL1*** | ***CCND1*** | ***CUL3*** | *ERCC4* | *GID4* | ***IDH2*** | ***LRP1B*** | ***NBN*** | ***PDGFRB*** | *PTPRS* | ***SDHC*** | ***TBX3*** | *ZBTB2* |
| *ASXL2* | ***CCND2*** | *CUL4A* | *ERCC5* | *GNA13* | *IFNGR1* | *LYN* | *NCOA3* | *PDK1* | *PTPRT* | ***SDHD*** | *TCF3* | *ZFHX3* |
| *ATF1* | ***CCND3*** | *CUL4B* | ***ERG*** | *GPS2* | *IGF1* | *LZTR1* | *NCOR1* | *PGR* | *QK1* | ***SETD2*** | *TCF7L2* | *ZNF217* |
| ***ATM*** | ***CCNE1*** | *CXCR4* | ***ERRFI1*** | ***GREM1*** | ***IGF1R*** | *MAG12* | *NEB* | *PHOX2B* | *RAB35* | ***SF3B1*** | ***TERC*** | *ZNF703* |
| ***ATR*** | ***CD274*** | *CYCLD* | ***ESR2*** | ***GRM3*** | ***IGF2*** | ***MALT1*** | *NEGR1* | ***PIK3CA*** | ***RAC1*** | *SH2B3* | ***TERT*** | *ZNRF3* |
| ***ATRX*** | *CD276* | *CYP17A1* | *EWSR1* | ***GSK3B*** | ***IKBKE*** | ***MAP2K1*** | ***NF1*** | ***PIK3CB*** | *RAD21* | *SH2D1A* | *TET1* | *ZRSR2* |
| ***AURKA*** | ***CD79A*** | ***DAXX*** | ***EZH2*** | ***GSTM1*** | ***IKZF1*** | ***MAP2K2*** | ***NF2*** | *PIK3C2B* | ***RAD50*** | *SHQ1* | ***TET2*** |  |
| ***AURKB*** | ***CD79B*** | *DCUN1D1* | ***FAM175A*** | ***GSTT1*** | *IL10* | ***MAP2K4*** | ***NFE2L2*** | *PIK3C2G* | ***RAD51*** | *SLIT2* | *TGFBR1* |  |
| ***AXIN1*** | ***CDC73*** | ***DDR2*** | ***FAM46C*** | ***H3F3A*** | ***IL7R*** | ***MAP3K1*** | ***NFKB1A*** | *PIK3C3* | ***RAD51B*** | ***SLX4*** | ***TGFBR2*** |  |
| *AXIN2* | ***CDH1*** | ***DICER1*** | ***FANCA*** | *H3F3B* | ***INHA*** | *MAP3K13* | ***NKX2-1*** | *PIK3CD* | ***RAD51C*** | ***SMAD2*** | *TIPARP* |  |
| ***AXL*** | ***CDK12*** | *DIS3* | ***FANCC*** | *HDAC1* | ***INHBA*** | *MAP3K14* | *NKX3-1* | ***PIK3CG*** | ***RAD51D*** | ***SMAD3*** | *TMEM127* |  |
| *B2M* | ***CDK4*** | *DNAJB1* | ***FANCD2*** | *HDAC2* | *INPP4A* | *MAP3K3* | ***NOTCH1*** | ***PIK3R1*** | ***RAD52*** | ***SMAD4*** | *TMPR552* |  |
| *BACH1* | ***CDK6*** | *DNMT1* | ***FANCE*** | *HDAC4* | ***INPP4B*** | *MAPK1* | ***NOTCH2*** | ***PIK3R2*** | ***RAD54L*** | ***SMARCA4*** | ***TNFAIP3*** |  |
| ***BAP1*** | ***CDK8*** | ***DNMT3A*** | ***FANCF*** | ***HGF*** | *INSR* | *MAX* | ***NOTCH3*** | *PIK3R3* | ***RAF1*** | ***SMARCB1*** | ***TNFRSF14*** |  |
| ***BARD1*** | ***CDKN1A*** | *DNMT3B* | ***FANCG*** | ***HIST1H1C*** | *IRF2* | ***MCL1*** | *NOTCH4* | *PIM1* | *RANBP2* | *SMARCD1* | ***TNFSF11*** |  |
| *BBC3* | ***CDKN1B*** | ***DOT1L*** | ***FANCI*** | ***HIST1H2BD*** | ***IRF4*** | ***MDM2*** | ***NPM1*** | ***PLCG2*** | ***RARA*** | ***SMO*** | ***TOP1*** |  |
| ***BCL2*** | ***CDKN1C*** | *E2F3* | ***FANCL*** | ***HIST1H3A*** | ***IRS1*** | ***MDM4*** | ***NRAS*** | *PLK2* | *RASA1* | *SNCAIP* | *TOP2A* |  |
| *BCL10* | ***CDKN2A*** | *EED* | *FANCM* | ***HIST1H3B*** | ***IRS2*** | ***MED12*** | *NR4A3* | *PMAIP1* | ***RB1*** | ***SOCS1*** | ***TP53*** |  |

Genes in bold denote the interrogation of whole exons. Genes in regular face denote the interrogation of critical exons, introns and promoter region. Blue shading denotes the detection of single nucleotide variations and copy number variations. Gray shading denotes detection of only single nucleotide variations. No shading denotes the detection of only fusions.
