# Supplementary material for: Genomic profiling of ovarian clear cell carcinoma in Chinese patients reveals potential prognostic biomarkers for survival
Source: Ann Med. 2023 Jun 5;55(1):2218104. doi: 10.1080/07853890.2023.2218104 (PMC10243386; doi:10.1080/07853890.2023.2218104)
Supplement: Supplemental Material [file IANN_A_2218104_SM2762.docx]

Supplementary Table S2. Next-generation sequencing studies in OCCC

| Author and Year | Sample | Size (n) | Patient location | Sequence | Stage |
| --- | --- | --- | --- | --- | --- |
| Friedlander et al 2016 [12]* | FFPE | 105 | Australia | 46-gene panel | NA |
| Wang et al 2017 [18] | Frozen tissue | 35 | Canada, Japan | Whole-genome sequencing | NA |
| Maru et al 2017 [16] | FFPE | 18 | Japan | 409-gene panel | I/II 15 (83.3%); III/IV 3 (16.7%) |
| Elvin et al 2017 [14] | FFPE | 125 | USA | 315-gene panel | NA |
| Arildsen et al 2017 [13] | FFPE | 10 | Sweden, Denmark | 60-gene panel | NA |
| Itamochi et al 2017 [15] | Frozen tissue | 55 | Japan | Whole-genome sequencing | I/II 33 (60%); III/IV 22 (40%) |
| Murakami et al 2017 [17] | Frozen tissue | 39 | Japan | Whole-exome sequencing | NA |
| Shibuya et al 2017 [21] | FFPE | 48 | Japan | Whole-exome sequencing | I/II 29 (60.4%); III/IV 19 (39.6%) |
| Kim et al 2018 [20] | Frozen tissue | 15 | Korea | Whole-exome sequencing | I/II 11 (73.3%); III/IV 4 (26.7%) |
| Caumanns et al 2018 [19] | Frozen tissue | 124 | Belgium, Germany, Norway, Poland, Netherlands, UK, USA | Kinome sequencing and 48 cancer-related genes | NA |
| Takenaka et al 2019 [22] | FFPE | 68 | Japan, Australia | 103-gene panel | I/II 19 (27.9%); III/IV 49 (72.0%) |
| Saotome et al 2020 [24] | Frozen tissue | 30 | Japan | 160-gene panel | I/II 24 (80%); III/IV 6 (20%) |
| Yang et al 2020 [25] | FFPE | 69 | China | Whole-exome sequencing (n=42); targeted sequencing (n=69) | I/II 57 (82.6%); III/IV 12 (17.4%) |
| Lapke et al 2021 [35] | FFPE | 23 | Taiwan, China | 410-gene panel | NA |
| Kuroda et al 2021 [12] | FFPE | 41 | Japan | 160-gene panel | I/II 32 (78.0%); III/IV 92 (22.0%) |
| Ye at al 2021 (our study) | FFPE | 61 | China | 520-gene panel | I/II 41 (67.2%); III/IV 19 (31.1%) |
| * Reference number.  Abbreviations: FFPE= Formalin-Fixed Paraffin-Embedding; NA=Not Available | | | | | |
