# Supplementary material for: Genomic profiling of ovarian clear cell carcinoma in Chinese patients reveals potential prognostic biomarkers for survival
Source: Ann Med. 2023 Jun 5;55(1):2218104. doi: 10.1080/07853890.2023.2218104 (PMC10243386; doi:10.1080/07853890.2023.2218104)
Supplement: Supplemental Material [file IANN_A_2218104_SM2761.docx]

Supplementary Table S1. Main findings of the genetic variations in this and previous studies of ovarian clear cell carcinoma (OCCC).

| Author and Year | Major findings |
| --- | --- |
| Friedlander et al 2016 [12]* | Molecular profiling was performed on pure (n = 105) and mixed (n = 26) OCCC patients based on histology. The most common findings were in the PIK3CA/Akt/mTOR pathway, with 61% cases showing a molecular alteration in one of these pathway components. Frequently altered genes included PIK3CA (50% in pure OCCC vs 18.5% in mixed), TP53 (18.1% vs 57.7%), KRAS (12.4% vs 3.7%), cMET (1.9% vs 11.1%), and PTEN (1.0% vs 7.7%). Altered HER2, PDGFRA, and NRAS was each identified in 1 pure OCCC case. |
| Wang et al 2017 [18] | The authors performed whole genome sequencing on 35 OCCC samples. Ab initio clustering revealed OCCC grouped according to APOBEC-related mutational process (26%) and age-related mutational signatures (40%). |
| Maru et al 2017 [16] | Targeted sequencing analysis 18 samples identified 45 somatic mutations in 34 genes. The most frequently altered genes included PIK3CA (28%), ARID1A (16.7%), CTNNB1 (11.1%), CSMD3 (11.1%), LPHN3 (11.1%), LRP1B (11.1%), and TP53 (11.1%). |
| Elvin et al 2017 [14] | A total of 125 cases, 45.6% from primary site tissue and 54.4% from metastatic sites (13.6% regional and 40.8% distant), were included. At least one clinically relevant genomic alteration was found in 112 (89.6%) case (average 2.2). The most frequently affected genes included PIK3CA (52.8%), ARID1A (51.2%), TP53 (21.6%), ZNF217 (17.6%), ERBB2 (12.8%), KRAS (8%), CCNE1 (7.2%), AKT2 (7.2%), FBXW7 (5.6%), CRKL (4.8%). The cohort had relatively lower rates of focal MET amplification (1.6%), PTEN loss (5.6%), and MSI-H status (4%). Eighty-seven (69.6%) patients harbored mutations in at least one member of the PI3K/Akt/mTOR pathway. Additionally, mutation of SWI/SNF complex gene SMARCA4 was identified in one case. |
| Arildsen et al 2017 [13] | Targeted sequencing was performed on 10 primary OCCCs and revealed 20 somatic non-synonymous variants or insertions/deletions in 11 genes: ARID1A (40%), PIK3CA (40%), TP53 (20%), ALK (10%), CDKN2A (10%), ERBB2 (10%), KMT2D (10%), KRAS (10%), NOTCH (10%), ROS1 (10%), SPOP (10%). Notably, 3 of these genes are involved in chromatin remodeling. |
| Itamochi et al 2017 [15] | Whole-genome sequencing of 55 samples identified alterations to the SWI/SNF complex subunit, PI3K/Akt and receptor tyrosine kinase (RTK)/Ras pathways were found in 51%, 42%, and 29% cases, respectively. Multivariable analysis suggested activated PI3K/Akt or RTK/Ras signaling pathways as independent prognostic factors for OCCC. |
| Murakami et al 2017 [17] | Whole exome sequencing was performed on 39 tumor tissue samples and identified 426 genes that were altered in >1 patients. Frequently aberrant genes included ARID1A (62%), PIK3CA (51%), MLL3 (15%), ARID1B (10%), KRAS (10%), PPP2R1A (10%), PIK3R1 (8%) and PTEN (5%). The authors also reported frequent amplification in chr8q (64%), chr20q (54%), and chr17q (46%) loci as well as deletion in chr19p (41%), chr13q (28%), chr9q (21%), and chr18q (21%) loci. The frequently mutated or amplified/deleted genes were involved in the KRAS/PI3K (82%) and MYC/retinoblastoma (75%) pathways as well as the SWI/SNF complex (85%). |
| Shibuya et al 2017 [21] | The authors applied whole exome sequencing on 48 OCCC tissues. The frequently mutated genes were ARID1A (66.7%), PIK3CA (50%), PPP2R1A (18.8%) and KRAS (16.7%). There were also somatic mutations with clinical implications, including those in BRAF (4.2%), ERBB2 (2.1%), PDGFRB (2.1%) and PGR (2.1%). The authors also found 3 (6.3%) hypermutated cases. Additionally, three clusters were identified based on mutation spectra, one of which characterized by APOBEC activation. |
| Kim et al 2018 [20] | Targeted sequencing was performed on 15 patients. A median of 178 exonic mutations (range, 111-25,798) and a median of 343 somatic copy number variations (range, 43-1,820) were found per tumor sample. In terms of mutations, 54 somatic mutations across 14 genes were detected, including those in PIK3CA (40%), ARID1A (40%), and KRAS (20%). Copy number gains in NTRK1 (33%), MYC (40%), and GNAS (47%) and copy number losses in TET2 (73%), TSC1 (67%), BRCA2 (60%), and SMAD4 (47%) were frequent. Functionally, the significantly altered pathways included PI3K/AKT, TP53, and ERBB2 pathways (collectively altered in 87% cases) and chromatin remodeling (47% cases). |
| Caumanns et al 2018 [19] | Targeted sequencing of 124 patients found that 91% patients harbored alterations in PI3K/AKT/mTOR pathway (PIK3CA: 44.3%, PTEN: 6.6%), PIK3R1: 7.4% and AKT1: 4.9%), the MAPK pathway (KRAS: 15.6%) or the ERBB family of receptor tyrosine kinases (ERBB3: 6.6%, EGFR: 4.1%) and that 82% patients had aberrant DNA repair pathway (TP53: 11.5%, ATM: 9%, PRKDC: 8.2%). Additionally, ARID1A mutations (54/116, 46.6%) were revealed using haloplex sequencing. Kinome sequencing also revealed altered kinase-encoding genes, including MAST4 (6.6%), TRRAP (5.7%), LRRK2 (5.7%), MYO3A (5.7%), WNK2 (4.9%), EIF2AK4 (4.9%), TAF1 (4.1%). A ubiquitin E4 ligase FBXW7 was also found altered in 4.1% cases. |
| Takenaka et al 2019 [22] | The authors performed targeted sequencing on 68 OCCC cases and exploratory whole exome sequencing on 10 of the 60 patients. Notably, most mixed OCCC (N=6, 85.7%), and a small proportion of pure OCCC (N=3, 4.9%) were re-classified as likely high-grade serous ovarian cancer. Among the 54 cases re-classified as OCCC, the frequently mutations included ARID1A (63.0%), PIK3CA (38.9%), PPP2R1A (14.8%), KRAS (13.0%) and TP53 (9.3%). However, none appeared associated with PFS or OS. |
| Saotome et al 2020 [24] | Targeted sequencing was performed on a cohort that included 30 OCCC patients. Druggable hypermutation (tumor mutation burden ≥ 10 SNVs/Mbp) was found in 10% OCCC cases. The frequently altered genes included ARID1A (30.0%), PIK3CA (26.7%), TP53 (13.3%), KRAS (6.7%), AKT1 (3.3%), PIK3R1 (3.3%), MSH6 (3.3%), BRCA2 (3.3%), CTNNB1 (3.3%), and MET (3.3%). A high count of copy number alteration was associated with worse survival in all histotypes. |
| Yang et al 2020 [25] | The investigators analyzed 42 OCCC samples with whole exome sequencing (WES) and 74 with targeted sequencing (TS). WES showed that the frequently mutated genes included ARID1A (64.3%), MUC4 (28.6%), PIK3CA (28.5%), MAGEE1 (19%), ARID3A (16.7%), PPP2R1A (11.9%), PTEN (7.1%), and KRAS (4.8%). Functional analysis revealed aberrant proliferation and survival pathways (including the PI3K/AKT, TP53, and ERBB2 pathways) in 83% cases and chromatin remodeling in 71% cases. Altered MAGEE1 (64% in the TS cohort) was associated with worse clinical outcomes. |
| Lapke et al 2021 [35] | The authors performed targeted sequencing on 23 OCCC cases along with other ovarian cancer histotypes. The frequently altered genes in OCCC included DNA repair-related genes ARID1A (39%), MLH1 (9%), MRE11A (4%), BLM (4%), ATM (4%), and BAP1 (4%), receptor tyrosine kinases ERBB2 (22%), MET (9%), IGFR1R (4%), and RET (4%), PI3K/AKT/mTOR pathway components PIK3CA (43%), AKT1 (9%), TSC1 (9%), TSC2 (4%), FBXW7 (9%), and STK11 (9%), RAS/MAPK pathway member KRAS (13%) and MAPK1 (9%), cell cycle genes CDKN2A (13%), CDKN2B (9%), CCND1 (4%), and CDK4 (4%), and hedgehog pathway gene PTCH1 (9%). |
| Kuroda et al 2021 [12] | The investigator performed targeted sequencing analysis on 41 OCCC patients along with other ovarian cancer histotypes. ARID1A mutation and loss were respectively found in 41.5% and 75.6% OCCC cases. ARID1A mutation and loss were both associated with high PD-L1 or CD8 levels. Also, ARID1A mutation was associated with high tumor mutation burden in OCCC. |
| Ye at al 2021 (our study) | Targeted sequencing of 50 patients showed that the most common mutant genes were ARID1A (50%) and PIK3CA (52%). Other frequently mutated genes included TP53 (18%), ATM (18%), ERBB2 (16%), SMARCA4 (14%), PRKDC (14%), PBRM1 (12%), LRP1B (12%), ARID5B (10%), GNAS (10%), PPP2R1A (10%), ATRX (8%), KRAS (8%), and CCNE1 (7%). Tumor mutation burden was low (average 4.36). |

* Reference number.
